# Epidemiological Profile and Mortality Patterns of Fatal Burn Injuries: A Retrospective Autopsy-Based Study From North India

**DOI:** 10.7759/cureus.95212

**Published:** 2025-10-23

**Authors:** Arushi Verma, R Sivasankary, Neha Soibam, Kishanth S, Yashpal S, Raviprakash Meshram, Shailesh V Parate

**Affiliations:** 1 Forensic Medicine and Toxicology, All India Institute of Medical Sciences, Rishikesh, Rishikesh, IND

**Keywords:** burn deaths, burns, flame burn, septicemia, thermal injury

## Abstract

Burn injuries constitute a significant cause of unnatural deaths in developing countries like India, particularly affecting women in domestic settings. This retrospective, autopsy-based study comprising 25 confirmed burn death cases from October 2018 to May 2025 (6 years and 7 months), was conducted at the Department of Forensic Medicine and Toxicology, AIIMS Rishikesh, a tertiary care hospital in North India. The objective of the study was to assess the epidemiological profile and autopsy findings of fatal burn cases for understanding patterns and determinants of burn-related mortality. In our study, the majority of victims were young adults aged 20-39 years, with a slight female predominance (56%, n=14). Most incidents occurred during winter (68%, n=17) and at home (76%, n=19), with flame burns being the most common type (84%, n=21). Accidental burns accounted for 80% (n=20) of cases, while suicidal and homicidal burns comprised 12% (n=3) and 4% (n=1), respectively. More than 68% of the cases had burns involving >40% of the total body surface area, while 32% (n=8) had burns involving >80% of the total body surface area. Survival was inversely related to total body surface area burnt, with more than 60% of victims dying within the first 7 days. Septicemia (76%, n=19) was the leading cause of death among burn victims. These findings underscore the need for improved domestic fire safety, early medical care, and better implementation of various government-run initiatives for the prevention and management of burn injuries.

## Introduction

Burns constitute a significant global public health concern, accounting for an estimated 180,000 deaths annually [1]. Most of these fatalities occur in low- and middle-income countries (LMICs), with nearly two-thirds reported from the WHO African and South-East Asia regions. Children under five years of age and elderly adults represent the most vulnerable groups affected by burn injuries in these countries [1]. Burns are also among the leading causes of disability-adjusted life years (DALYs) lost in LMICs. Globally, burns are the fourth most common cause of trauma, following road traffic accidents, falls, and interpersonal violence [2].

In India, the absence of a centralized national burn registry limits the availability of accurate epidemiological data on burn injuries. According to the National Crime Records Bureau (NCRB), an estimated 7,435 deaths due to fire accidents were reported in 2022, reflecting a significant decline from 27,561 deaths recorded in 1996. On average, women accounted for approximately 60% of all fire-related fatalities. While the NCRB data indicate a downward trend in fire-related deaths, the reliability of these figures warrants cross-verification with hospital records, fire department data, and existing burns registries to ensure accuracy [3]. Estimates extrapolated from three major government hospitals in Delhi suggest that around 140,000 people die annually from burn injuries--equivalent to 1 death every 4 minutes. Furthermore, the estimated annual incidence of burn injuries in India is approximately six to seven million cases [4].

The increased vulnerability among females is often associated with unsafe cooking practices, domestic violence, and dowry-related burn incidents. Major risk factors for burns include the use of open-fire cooking, kerosene stoves, poverty, overcrowded living conditions, loose-fitting clothing, and inadequate safety infrastructure [1,2].

The present study is a retrospective, autopsy-based study of 25 confirmed burn death cases brought for medico-legal autopsy at the Advanced Autopsy Center, AIIMS Rishikesh. The present study aims to examine the demographic distribution, manner of death, type and extent of burns, seasonal trends, and the underlying cause of death in these cases. Through this analysis, we seek to elucidate critical epidemiological patterns and autopsy findings in burn fatalities. The insights derived may serve to strengthen medico-legal investigations, inform public health interventions, and contribute to the broader forensic and epidemiological understanding of burn-related mortality.

## Materials and methods

Type of study

This was a retrospective, autopsy-based descriptive study.

Study objective

The objective of this study was to assess the epidemiological profile and autopsy findings of fatal burn cases to understand the patterns and determinants of burn-related mortality.

Study period

The study spanned from October 2018 to May 2025 (6 years and 7 months).

Study area

The study was conducted at the Department of Forensic Medicine and Toxicology, AIIMS Rishikesh, a tertiary care hospital in North India.

Study population

All burn death cases brought for a medico-legal autopsy at the Advanced Autopsy Center of the hospital during the study period constituted the population.

Study sample

A total of 25 confirmed burn death cases were brought for medico-legal autopsy to the hospital during the study period, and all were included in the study, as all met the inclusion criteria.

Inclusion Criteria

All burn death cases brought for a medico-legal autopsy to the Advanced Autopsy Center of the hospital during the study period, as per case records, were included.

Exclusion Criteria

All case records were studied, and cases that did not sustain any burn injuries and cases with electrical injury were excluded.

Data collection

Data were collected from autopsy registers, inquest papers, postmortem reports, and hospital case records (when available) by a team of three resident doctors of the department. A detailed data abstraction form was prepared to get the information from the records, which included the age and sex of the deceased; the manner and type of burn (e.g., flame, scald, etc.); the season and location of the burn incident; the percentage of total body surface area (TBSA) involved; the duration of survival following the injury; and the cause of death.

Data analysis

All data from the data abstraction form was entered into a Microsoft Excel sheet (Microsoft Corporation, Redmond, WA, US). From the Microsoft Excel sheet, the data were analyzed using IBM SPSS Statistics for Windows, Version 23.0 (Released 2015; IBM Corp., Armonk, NY, USA). Percentages were calculated and compared with other similar studies. Data were presented using tables and graphs. No inferential statistics were applied due to the limited sample size.

Ethical clearance

Ethical clearance for the study was obtained from the Institutional Ethics Committee of AIIMS Rishikesh (Reg. No.: EC/NEW/Inst/2022/UA/0180). Since the study involved the use of official autopsy and hospital case records without any interaction with living subjects, the requirement for informed consent was waived, as per national ethical guidelines on research involving the dead. Confidentiality of all identifying personal information was strictly maintained throughout the data collection and analysis process.

## Results

Out of a total of 4,181 medico-legal autopsies conducted during the study period, 25 cases (0.60%) were attributable to burn injuries.

Age and sex distribution

The data indicate that the majority of victims were aged between 20 and 39 years, with the 30-39 age group accounting for 32% (n = 8) of all cases. A modest female preponderance was observed, with 56% (n=14) females versus 44% (n=11) males, and the female deaths were distributed across both younger and middle-aged groups (Table 1).

**Table 1:** Age and sex distribution of burn deaths

Age group (years)	Male (n, %)	Female (n, %)	Total (n)
0–19	2 (8%)	1 (4%)	3
20–29	2 (8%)	2 (8%)	4
30–39	3 (12%)	5 (20%)	8
40–59	1 (4%)	3 (12%)	4
>= 60	3 (12%)	3 (12%)	6
Total	11 (44%)	14 (56%)	25

Seasonal and seasonal variation

**Figure 1:**
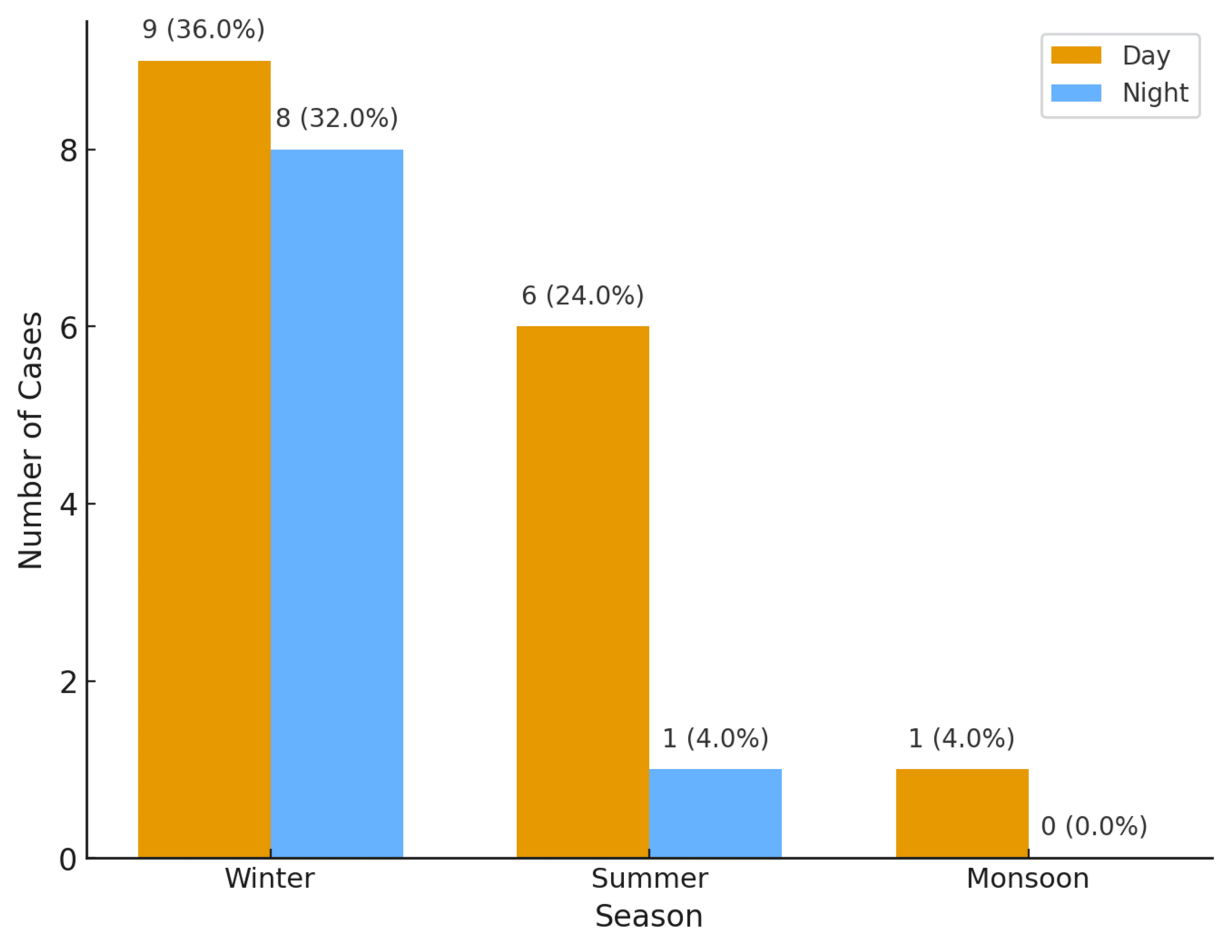
Seasonal and diurnal variation of burn deaths

A striking 68% (n=17) of deaths occurred during winter, with 36% (n=9) occurring during the day and 32% (n=8) during the night, highlighting a seasonal spike in burn incidents (Figure 1). Morning hours (44%, n=11) were the most common time of the incident.

Location and type of burn

**Figure 2:**
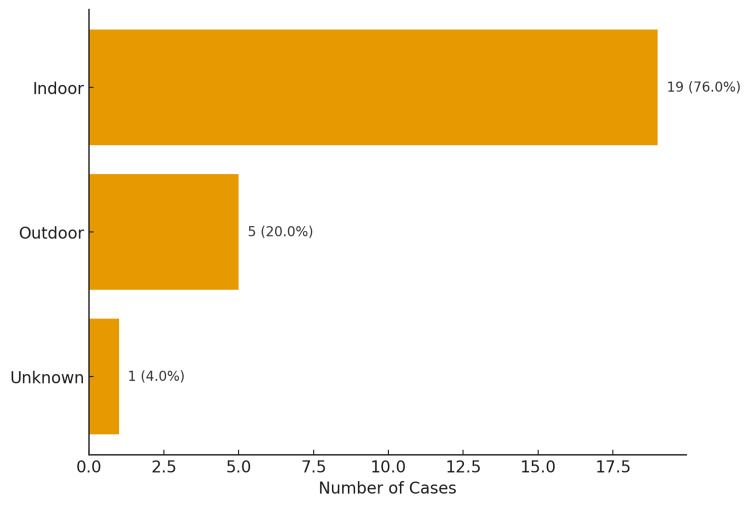
Location of burn deaths

**Figure 3:**
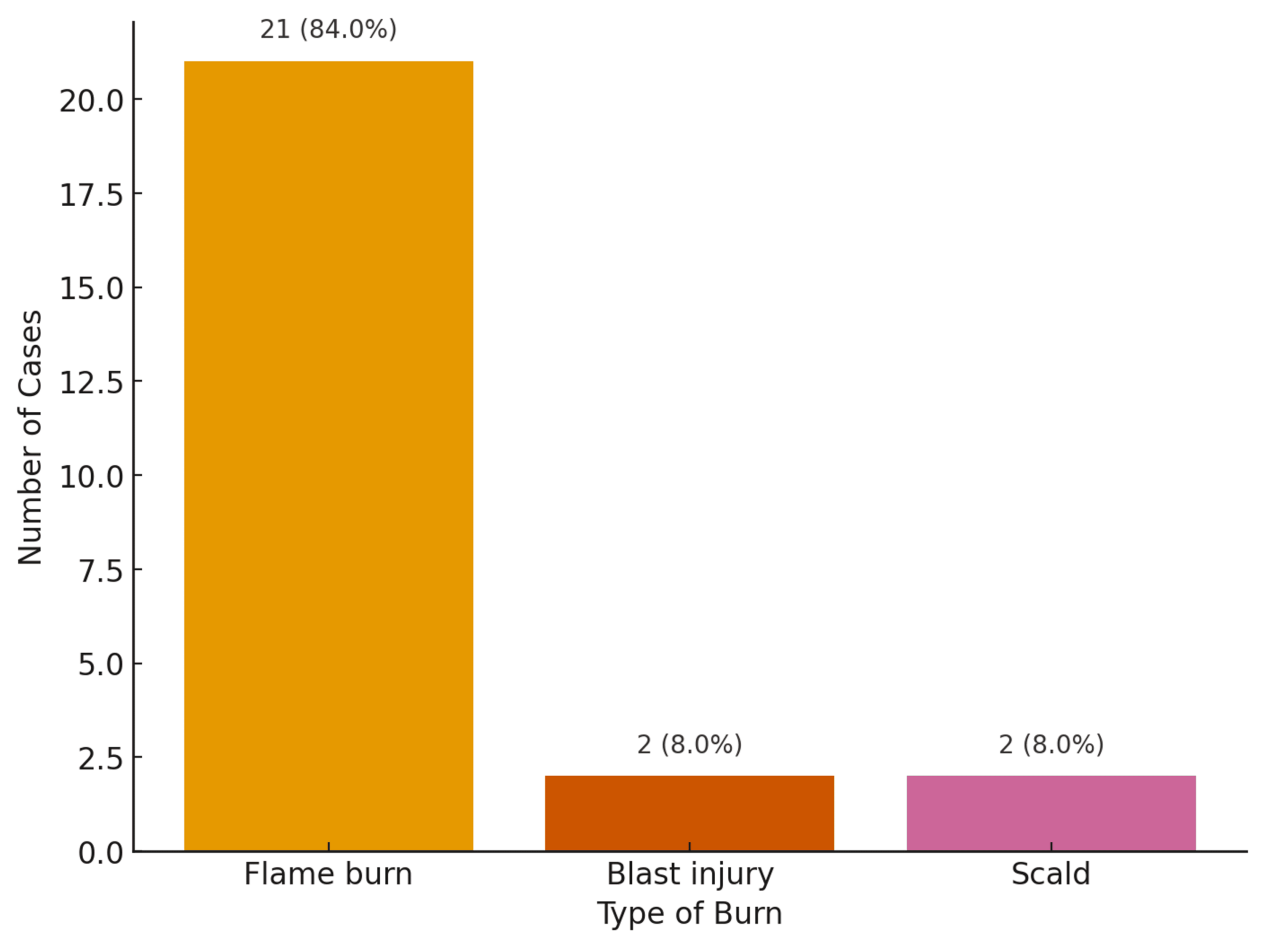
Type of burn

Most incidents occurred indoors, at home (76%, n=19), reinforcing that domestic environments are still the primary setting for fatal burn injuries. Twenty percent (20%; n=5) occurred outside, and the location of incidence was unknown in 4% (n=1) of cases (Figure 2). In terms of etiology, flame burns were overwhelmingly dominant (84%, n=21), while blast injuries (8%, n=2) and scalds (8%, n=2) were minor contributors (Figure 3).

Manner of death

Accidental burns accounted for the majority of deaths (80%, n=20), with a notable female preponderance--44% (n=11) of the total cases were females and 36% (n=9) were males. Suicidal burns were reported in 12% (n=3) of cases, predominantly among males (8%, n=2) as compared to females (4%, n=1). Homicidal burns were seen in 4% (n=1) of cases, and the manner was unknown in 4% (n=1) of cases. These findings highlight accidental burns as the most frequent manner of death (Table 2).

**Table 2:** Manner of burn deaths in relation to sex

Manner	Male (n,%)	Female (n,%)	Total
Accidental	9 (36%)	11 (44%)	20 (80%)
Homicidal	0 (0%)	1 (4%)	1 (4%)
Suicidal	2 (8%)	1 (4%)	3 (12%)
Unknown	0 (0%)	1 (4%)	1 (4%)

Total body surface area (TBSA%) burnt

The extent of TBSA burned was a crucial determinant of outcome. More than 68% of victims had > 40% TBSA burnt. Thirty-two percent (32%; n=8) of victims had burns exceeding 80% TBSA. The distribution of cases peaked in the 40-59% TBSA range.

**Figure 4:**
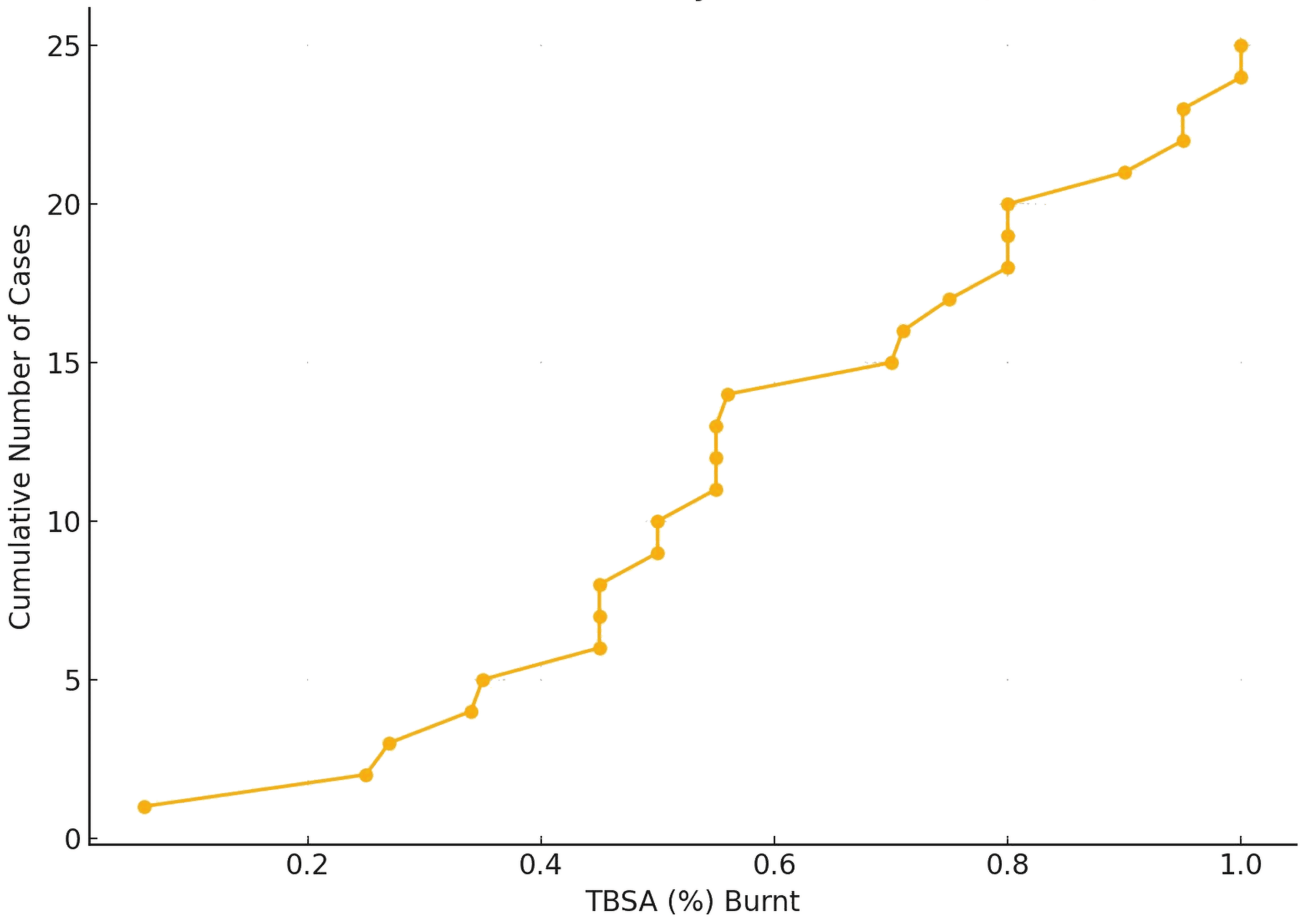
Cumulative plot of body surface area burnt

The cumulative TBSA plot demonstrates that nearly all fatal burn cases involved patients with more than 40% TBSA involvement. This finding highlights a critical threshold beyond which the risk of mortality increases sharply (Figure 4).

Survival period

More than 60% of deaths occurred within the first 7 days, particularly in the 4-7 day window (28%, n=7). Twenty percent (20%; n=5) died within 24 hours, and 16% (n=4) survived for >14 days. The 4-7 day survival group constituted the largest proportion of deaths, with a mean TBSA of 70.7%. Those who died within 24 hours had the highest mean TBSA (82.2%). Patients surviving more than 14 days had a significantly lower mean TBSA (25.8%), supporting the conclusion that lower TBSA is associated with prolonged survival (Table 3).

**Table 3:** Total body surface area burned in relation to the survival period Mean TBSA (%) = Σ TBSAᵢ / n (computed within each survival-period category), where TBSAᵢ is the TBSA (%) for case i and n is the number of cases in that category; values rounded to one decimal place. TBSA: total body surface area

Survival Period (days)	Number of Cases (n, %)	Mean TBSA (%)
1–3	4 (16%)	62.7
4–7	7 (28%)	70.7
8–14	5 (20%)	51.8
<1	5 (20%)	82.2
>14	4 (16%)	25.8

Cause of death

**Figure 5:**
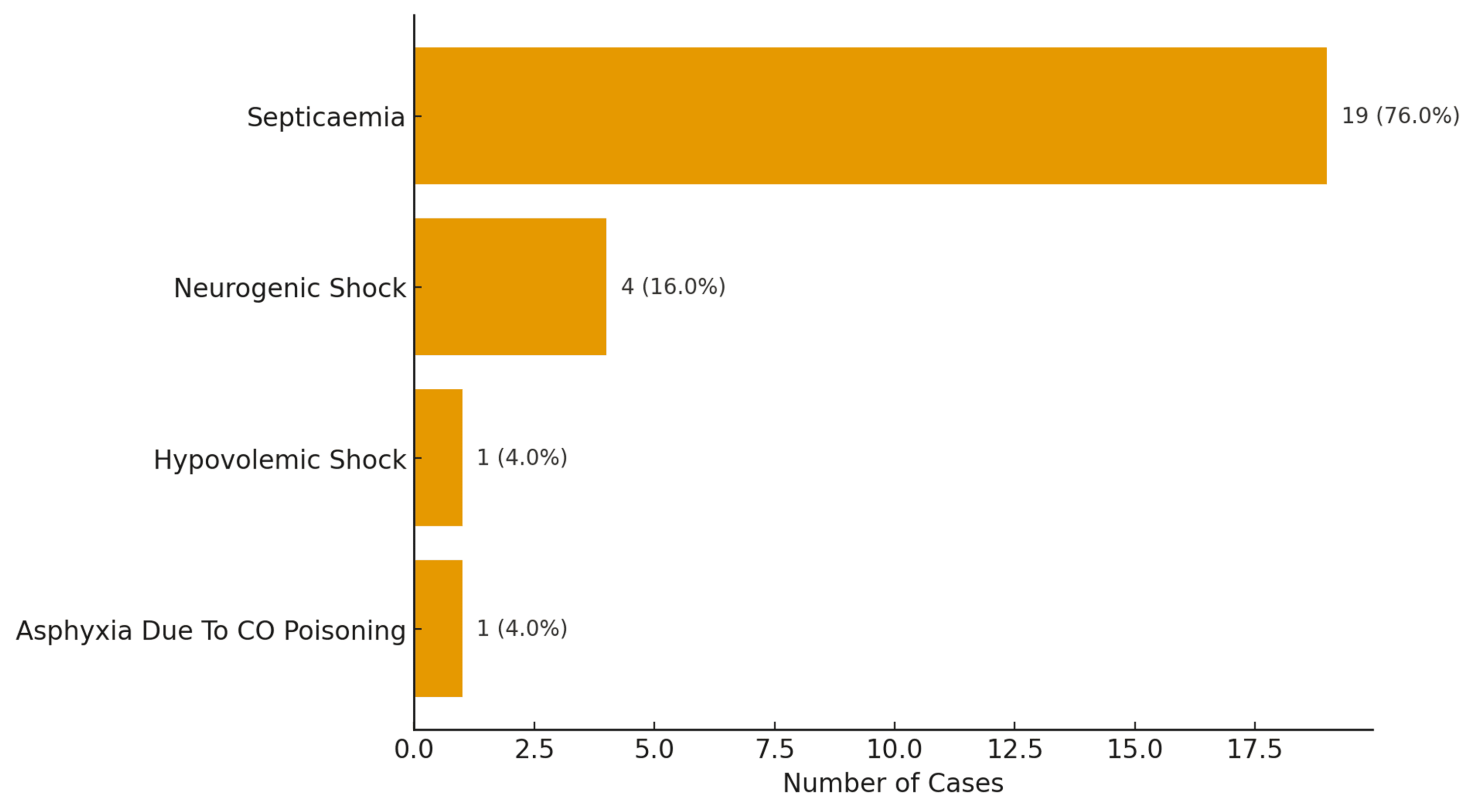
Cause of death in burn deaths

Septicemia was the most frequent cause of death, accounting for 76% (n=19) of all fatalities, followed by neurogenic shock (16%, n=4) (Figure 5).

## Discussion

The present autopsy-based study offers valuable insight into the epidemiological and medico-legal characteristics of burn fatalities in a tertiary care setting in North India. The findings are broadly consistent with previous Indian and global literature, although certain deviations were noted, suggesting regional, infrastructural, and socio-cultural influences.

In our study, young adults (20-39 years) were most affected (48%, n=12), with a slight female predominance (56%, n=14), broadly consistent with other Indian studies [5,6]. By contrast, research from some regions reports a higher proportion of male burn victims [7-9], highlighting that gender distribution varies by age, region, and national income category. WHO data show that in high-income countries, fire-related deaths among males aged 15-59 years are twice as high as in females, whereas in low-income countries, females in the same age group die from burns at 2.3 times the male rate [10]. Most international studies report a higher incidence among boys during childhood, regardless of a country’s level of development [11,12]. In developing countries, women are disproportionately affected due to domestic roles, socio-cultural hazards, such as dowry-related violence, and the use of loose, flammable clothing. In contrast, in developed countries, male predominance is linked to occupational exposure in high-risk jobs such as mining, firefighting, trucking, and aviation [2].

Our study observed that a substantial proportion of burn deaths (68%, n=17) occurred during the winter season, most frequently in the morning hours (44%, n=11). These findings are in agreement with earlier reports by Kumar et al., Singh et al., and Subrahmanyam et al. [5,6,13]. The higher incidence of burns in winter is closely related to seasonal practices, including the use of heating appliances, open wood fires, and kerosene heaters, in regions with unreliable or absent electricity supply. Additionally, winter mornings are associated with increased use of hot water for bathing, cooking, and other domestic chores, which further elevates the risk of accidental scald burns [14,15]. In contrast, studies from China and Turkey have documented a higher frequency of burn injuries in the summer months [16,17], reflecting how climatic conditions and regional lifestyle practices strongly influence the temporal distribution of burn cases.

The majority of burns occurred indoors at home (76%, n=19), a finding consistent with other major Indian and global studies [18-20]. Burns among adult females and children (0-14 years) predominantly occur at home, whereas burns in adult males are more frequently reported outdoors or at the workplace [21,22]. Workplace-related burns are more common in adult males because they are more likely to be employed in high-risk occupations such as laborers, electricians, and similar roles. Men also tend to sustain burns outdoors, particularly electrical injuries from power lines and transformer stations [2]. In children, most burns occurred at home, usually due to hot liquids and vapor [20,23]. Women, on the other hand, suffer most burns in domestic settings due to their household roles, especially those involving cooking and exposure to heating devices [2].

Flame burns accounted for 84% (n=21) of cases in our study. This finding is in line with other similar studies [5,6,11,13,19]. However, in studies involving pediatric populations, the overall major burns are from scalds, and over 50% of these are associated with food preparation or consumption, with a smaller proportion associated with bathing [20,23]. In our series, accidental deaths were the most common (80%, n=20), consistent with other studies [5,6,11,18,19,23]. However, Kumar S et al. (2013) reported suicidal burns to be slightly higher (38.6%) than accidental burns (37.3%) [13].

Our study showed that more than 68% of burn death cases involved >40% TBSA, and survival was inversely related to the extent of TBSA burnt. This parallels the findings of Kumar S et al. (2013) and Subrahmanyam M (1996), where the majority of victims had >50% and >40% TBSA involvement, respectively [13,6]. Similarly, an 80% mortality rate in burns involving 40-50% TBSA has been reported in other studies [19,24].

We also observed that most burn victims (>60%) died within seven days of admission, which is consistent with the findings of other studies [5,13,24]. Those who died within 24 hours had the highest mean TBSA (82.2%), while patients surviving beyond 14 days had a significantly lower mean TBSA (25.8%). These comparisons confirm that high TBSA is a critical predictor of burn mortality. Septicemia was identified as the leading cause of death in our series (76%, n=19), similar to findings in other studies [5,13,24]. These results underscore infection as the predominant terminal event in burn victims, particularly in those surviving the acute phase.

The present study has several limitations. First, it is a single-center, retrospective autopsy-based study, with a small sample size, which precluded inferential statistics and limits generalizability beyond our institutional catchment area. Second, the variables collected were restricted to demographic details, burn characteristics (type, location, season, TBSA), survival duration, and cause of death; important covariates, such as time-to-care, inhalational injury, co-morbidities, microbiology, and socioeconomic context, were not available, limiting causal inference. Finally, electrical injuries were excluded, narrowing mechanism coverage.

## Conclusions

The present study highlights the persistent burden of burn-related fatalities in low and middle-income countries like India, predominantly affecting young females in domestic settings. Most cases involved flame burns with high TBSA, and septicemia emerged as the leading cause of death. These findings reflect the urgent need for focused preventive strategies and timely medical intervention. Effective burn prevention requires public education on fire safety, safer cooking practices, and improved household infrastructure, particularly in rural areas. Gender-based violence and dowry-related burn deaths must be addressed through stringent legal measures and community-level awareness. Government-run programs like the National Programme for Prevention and Management of Burn Injuries (NPPMBI) are steps in the right direction, but demand better implementation, wider coverage, and dedicated burn care units at peripheral hospitals. The study highlights the importance of a multidisciplinary approach, involving public health awareness programs, medico-legal investigation, healthcare, and community engagement to reduce the incidence and mortality associated with burn injuries in India.
